# Frequency of Hepatitis B and Hepatitis C Infections Among Patients With Chronic Kidney Disease: A Retrospective Data-Based Study From Lahore, Pakistan

**DOI:** 10.7759/cureus.113515

**Published:** 2026-07-28

**Authors:** Muhammad Talha Sami, Ahtisham Liaqat, Muhammad Tayyab Latif, Naeem Akram, Aazia Sami, Nauman Ismat Butt

**Affiliations:** 1 Medicine, Azra Naheed Medical College, Superior University, Lahore, PAK; 2 Medicine and Rheumatology, Azra Naheed Medical College, Superior University, Lahore, PAK

**Keywords:** chronic kidney disease (ckd), hd (hemodialysis), hepatitis b virus (hbv), hepatitis c (hcv) infection, pakistan

## Abstract

Background and objective

Chronic hepatitis B virus (HBV) and chronic hepatitis C virus (HCV) infections are significant global public health challenges, affecting millions of individuals worldwide and contributing substantially to morbidity and mortality due to chronic liver disease and its complications. In Pakistan, the burden of viral hepatitis remains considerable, with HBV and HCV infections continuing to affect vulnerable populations and placing an additional healthcare burden on patients with chronic diseases, including chronic kidney disease (CKD). This study aimed to determine the frequency of both HBV and HCV infections separately among CKD patients and to evaluate their relationship with demographic and clinical factors in a local Pakistani population.

Methods

This retrospective data-based study was conducted at Chaudhary Muhammad Akram Teaching and Research Hospital (Lahore, PAK) and included 317 medical records of CKD patients aged ≥18 years who presented to the Department of Medicine and Nephrology for dialysis and ICU admission from January to December 2025. Incomplete medical records, patients with acute kidney injury, and records missing hepatitis serologies were excluded. A structured pro forma was used to extract demographic characteristics, CKD-related variables, and laboratory parameters including HBsAg and anti-HCV antibodies from patient medical records. Data were analyzed using SPSS Statistics version 23 (IBM Corp., Armonk, NY, USA).

Results

Of the 317 CKD patients, 185 (58.4%) were male, and 184 (58.0%) resided in urban areas. Mean CKD duration was 2.9±2.8 years, with 185 (58.4%) having CKD for ≥2 years and 246 (77.6%) with stage 5 CKD. Hypertension was present in 271 (85.5%), diabetes in 171 (53.9%), and cardiovascular disease in 72 (22.7%). History of blood transfusion was reported in 140 (44.2%), while 214 (67.5%) had a history of dialysis. The mean hemoglobin (Hb) was 9.3±1.5 g/dL, with 247 (77.9%) having Hb>8 g/dL. Hepatitis B surface antigen (HBsAg) positivity was detected in 13 (4.1%) and anti-HCV in 131 (41.3%). Five (1.5%) had both HBsAg and anti-HCV positivity. Chi-square analysis revealed significantly associated anti-HCV positivity with the male gender (p<0.001), urban residence (p=0.038), CKD duration ≥2 years (p=0.027), advanced CKD stage (p<0.001), cardiovascular disease (p=0.049), blood transfusion history (p<0.001), and dialysis history (p<0.001). Binary logistic regression analysis demonstrated male gender (OR=0.483, 95% CI: 0.277-0.839; p=0.010), urban residence (OR=0.520, 95% CI: 0.305-0.887; p=0.016), positive history of blood transfusion (OR=0.348, 95% CI: 0.183-0.660; p=0.001), and dialysis history (OR=0.211, 95% CI: 0.091-0.488; p<0.001) to be predictors of anti-HCV positivity. Fisher's exact test demonstrated a significant association of HBsAg with the female gender (p*=*0.047) and diabetes (p<0.001). Regression analysis was not applied for HBsAg due to the limited number of participants with HBV positivity.

Conclusion

Hepatitis C virus was highly prevalent in CKD patients; HBV remained comparatively uncommon. This emphasizes the need for routine screening among CKD patients, especially those undergoing dialysis or receiving blood transfusions. Strengthening infection-control measures, ensuring safe transfusion practices, promoting HBV vaccination, and providing timely antiviral management may reduce the burden of viral hepatitis and improve clinical outcomes in this high-risk population.

## Introduction

Chronic infections caused by hepatitis B virus (HBV) and hepatitis C virus (HCV) continue to impose a major global health burden, leading to substantial liver-related morbidity and mortality across diverse populations [1,2,3]. In Pakistan, these infections remain highly prevalent, particularly among individuals with chronic kidney disease (CKD), where they contribute to increased healthcare demands and adverse clinical outcomes [2,4]. Chronic kidney disease is characterized by a progressive and irreversible decline in renal function, leading to significant systemic complications including anemia, cardiovascular disease, hypertension, electrolyte disturbances, and mineral bone disorders in addition to mental distress, anxiety, depression, and an overall reduced life quality [5,6]. Patients with CKD, particularly those requiring hemodialysis, are at increased risk of acquiring blood-borne infections due to repeated vascular access procedures, frequent hospital exposure, blood transfusions, and prolonged healthcare contact [4,7].

The association between chronic viral hepatitis and renal dysfunction has been increasingly recognized. Persistent HBV and HCV infections may contribute to kidney injury through immune-mediated mechanisms, including glomerular deposition of immune complexes, leading to proteinuria, glomerulonephritis, and progressive deterioration of renal function [7,8]. Additionally, CKD patients with chronic hepatitis infections may experience increased morbidity due to accelerated disease progression and complications related to both renal and hepatic dysfunction. A multicenter study conducted in Pakistan reported the prevalence of HBV and HCV among CKD patients on hemodialysis as 5.4% and 28.8%, respectively, with 3.4% having HBV/HCV co-infection [4]. These findings highlight the substantial burden of viral hepatitis among patients receiving renal replacement therapy. However, most available local studies have focused primarily on dialysis populations, while limited data exist regarding the frequency of HBV and HCV infections among patients across different stages of CKD, including those not yet requiring dialysis [9,10,11].

Early identification of hepatitis infections among CKD patients is essential for implementing appropriate infection-control measures, optimizing treatment strategies, and reducing transmission risk, particularly in dialysis settings. Therefore, this study aims to determine the frequency of both chronic hepatitis B and C infections separately among CKD patients and to evaluate their relationship with demographic characteristics and CKD-related clinical factors in a local population.

## Materials and methods

This retrospective data-based observational study was conducted at the Departments of Medicine and Nephrology, Chaudhary Muhammad Akram Teaching and Research Hospital, affiliated with Azra Naheed Medical College, Superior University (Lahore, PAK). Data were collected after approval from the Institutional Ethical Review Committee of Azra Naheed Medical College (approval no. ANMC/IRB/2025/045). Patient confidentiality was maintained throughout the study, and personal identifiers were removed from the dataset. Data were accessed only by the research team and were stored securely.

Keeping a margin of error of 5% and a confidence interval of 95%, a sample size of 317 patients was calculated through the OpenEpi online calculator [12], using expected frequencies of hepatitis B and hepatitis C as 5.4% and 28.8%, respectively [4]. This retrospective study included medical records of adult patients aged ≥18 years diagnosed with CKD who presented to the Departments of Medicine and Nephrology, including patients attending the dialysis unit and admitted to the ICU, from January to December 2025. Chronic kidney disease was defined by an estimated glomerular filtration rate (eGFR) <60 mL/min/1.73 m² for at least three months along with ultrasonographic findings such as reduced kidney size, cortical thinning, increased cortical echogenicity, and/or the presence of persistent albuminuria. Based on eGFR, CKD was categorized as stage 3 (eGFR between 59 and 30 mL/min/1.73 m²), stage 4 (eGFR between 29 and 15 mL/min/1.73 m²), and stage 5 (eGFR less than 15 mL/min/1.73 m²). Incomplete medical records, records of patients with acute kidney injury, and records missing hepatitis serology results were excluded from the study.

A total of 317 records were included in the study using the non-probability consecutive sampling technique. Data were extracted from patient medical records using a structured data collection pro forma by five trained investigators working as a team. To ensure data accuracy, all extracted data were independently reviewed and cross-checked for completeness and consistency before entry into the study database. Any discrepancies were resolved through review of the original medical records. Information regarding demographic characteristics, including age, gender, and residence, was collected along with clinical CKD-related parameters such as duration of CKD, CKD stage according to eGFR, dialysis history, and history of blood transfusions. Relevant comorbid conditions, including diabetes, hypertension, and cardiovascular disease, were also recorded. Laboratory parameters included eGFR, serum creatinine, serum urea, serum sodium, serum potassium, hemoglobin (Hb) level, hematocrit, and platelet count.

The medical records were then assessed for hepatitis B and C infection. Hepatitis serological testing was performed as part of routine hospital care using standard enzyme-linked immunosorbent assay (ELISA) methods according to the laboratory's established protocols. Because of the retrospective nature of the study, information regarding the specific ELISA kit manufacturer and generation was not consistently available in the medical records. Hepatitis B infection was assessed by detecting hepatitis B surface antigen (HBsAg) using ELISA, according to standard laboratory protocols. Hepatitis C infection was evaluated through detection of anti-HCV antibodies performed using ELISA according to standard laboratory protocols. Patients with positive results were classified as having evidence of hepatitis infection.

Data were entered and analyzed using SPSS Statistics version 23 (IBM Corp., Armonk, NY, USA). Continuous variables were presented as mean ± standard deviation, while categorical variables were expressed as frequencies and percentages. Age and CKD duration were categorized into two groups based on 50% cumulative frequency (median). Anemia was defined as Hb <13.0 g/dl for males and Hb <12.0 g/dl for females. Severity of anemia was defined as severe if Hb was ≤8.0 g/dl and mild-to-moderate if Hb was >8.0 g/dl. Associations between hepatitis status and patient variables were assessed using Pearson's chi-square test when no more than 20% of the expected cell frequencies were <5% and all expected cell frequencies were ≥1%; otherwise, Fisher's exact test was applied. A p-value <0.05 was considered statistically significant. 

Subsequently, binary logistic regression analysis was performed to identify factors independently associated with anti-HCV status after adjustment for potential confounders. For regression analysis, anti-HCV status was entered as the dependent variable, while gender, age (years), residence, CKD duration, CKD severity, diabetes, hypertension, cardiovascular disease, history of blood transfusion, history of dialysis, and Hb level were entered as independent variables using the enter method. A two-tailed p<0.05 was considered statistically significant. For HBsAg, regression analysis tests were not applied due to the limited number of participants positive for HBV.

## Results

Among the 317 CKD patients, 185 (58.4%) were male, and 184 (58.0%) resided in urban areas (Table 1). The mean age was 51.0±13.0 years, with 164 (51.7%) patients aged ≥52 years. The mean CKD duration was 2.9±2.8 years, with 185 (58.4%) patients having CKD for ≥2 years. Mean eGFR was 11.6±10.5 ml/min/1.73 m³, with mean serum creatinine and serum urea at 7.2±3.2 mg/dL and 132.9±53.8 mg/dL, respectively. Based on eGFR, the majority of patients had stage 5 CKD (246, 77.6%), as shown in Table 1. Mean serum sodium was 136.7±3.0 mEq/L, while mean serum potassium was 4.8±0.6 mEq/L. Hypertension was present in 271 (85.5%) patients, followed by diabetes in 171 (53.9%) and cardiovascular disease in 72 (22.7%). A history of blood transfusion was reported in 140 (44.2%) patients, while 214 (67.5%) had a history of dialysis. All 317 (100%) patients had anemia with a mean Hb level of 9.3±1.5 g/dL, while 247 (77.9%) patients had Hb >8 g/dL. Mean hematocrit was 28.4±4.9%, while mean platelet count was 222.6±66.4×10⁹/L. Thirteen (4.1%) patients tested HBsAg positive, whereas anti-HCV antibodies were positive in 131 (41.3%) patients. Five (1.5%) patients were positive for both HBsAg and anti-HCV antibodies, indicating HBV/HCV co-infection.

**Table 1:** Patient variables (total n=317) CKD: Chronic kidney disease; HBsAg: Hepatitis B surface antigen; HCV: Hepatitis C virus; Hb: Hemoglobin

Patient variables		Frequency n (%)
Gender	Female	132 (41.6%)
	Male	185 (58.4%)
Age	<52 years	153 (48.3%)
	≥52 years	164 (51.7%)
Residence	Rural	133 (42.0%)
	Urban	184 (58.0%)
CKD duration	<2 years	132 (41.6%)
	≥2 years	185 (58.4%)
CKD severity	Stage 3	22 (6.9%)
	Stage 4	49 (15.5%)
	Stage 5	246 (77.6%)
Diabetes	Present	171 (53.9%)
	Absent	146 (46.1%)
Hypertension	Present	271 (85.5%)
	Absent	46 (14.5%)
Cardiovascular disease	Present	72 (22.7%)
	Absent	245 (77.3%)
Blood transfusion history	Absent	177 (55.8%)
	Present	140 (44.2%)
Dialysis history	Absent	103 (32.5%)
	Present	214 (67.5%)
Hb	≤8 g/dl	70 (22.1%)
	>8 g/dl	247 (77.9%)
HBsAg	Positive	13 (4.1%)
	Negative	304 (95.9%)
Anti-HCV	Positive	131 (41.3%)
	Negative	186 (58.7%)

As shown in Table 2, Fisher's exact test demonstrated a statistically significant association between gender and HBsAg status, with females having a higher prevalence of HBsAg positivity than males (6.8% vs. 2.2%; p=0.047). A significant association was also observed between diabetes and HBsAg status, as HBsAg positivity was found only among diabetic patients (7.6%, p<0.001). No statistically significant associations were observed between HBsAg status and age (p=0.876), residence (p=0.402), duration of CKD (p=0.596), CKD severity (p=0.598), hypertension (p=0.228), cardiovascular disease (p=0.180), history of blood transfusion (p=0.572), dialysis history (p=0.235), or Hb level (p=0.930).

**Table 2:** Stratification of data with regards to HBsAg (total n=317) CKD: Chronic kidney disease; HBsAg: Hepatitis B surface antigen; Hb: Hemoglobin

Patient variables		HBsAg		p-value
		Negative	Positive	
Gender	Female	123 (93.2%)	9 (6.8%)	0.047
	Male	181 (97.8%)	4 (2.2%)	
Age	<52 years	147 (96.1%)	6 (3.9%)	0.876
	≥52 years	157 (95.7%)	7 (4.3%)	
Residence	Rural	126 (94.7%)	7 (5.3%)	0.402
	Urban	178 (96.7%)	6 (3.3%)	
CKD duration	<2 years	128 (97.0%)	4 (3.0%)	0.596
	≥2 years	176 (95.1%)	9 (4.9%)	
CKD severity	Stage 3	22 (100%)	0 (0.0%)	0.598
	Stage 4	47 (95.9%)	2 (4.1%)	
	Stage 5	235 (95.5%)	11 (4.5%)	
Diabetes	Present	158 (92.4%)	13 (7.6%)	<0.001
	Absent	146 (100%)	0 (0.0%)	
Hypertension	Present	258 (95.2%)	13 (4.8%)	0.228
	Absent	46 (100%)	0 (0.0%)	
Cardiovascular disease	Present	67 (93.1%)	5 (6.9%)	0.180
	Absent	237 (96.7%)	8 (3.3%)	
Blood transfusion history	Absent	171 (96.6%)	6 (3.4%)	0.572
	Present	133 (95.0%)	7 (5.0%)	
Dialysis history	Absent	101 (98.1%)	2 (1.9%)	0.235
	Present	203 (94.9%)	11 (5.1%)	
Hb	≤8 g/dl	67 (95.7%)	3 (4.3%)	0.930
	>8 g/dl	237 (96.0%)	10 (4.0%)	

As presented in Table 3, Pearson's chi-square analysis revealed that anti-HCV positivity was significantly associated with male gender (51.9% vs. 26.5% in females; p<0.001), urban residence (46.2% vs. 34.6% in rural residents; p=0.038), CKD duration of ≥2 years (46.5% vs. 34.1%; p=0.027) and advanced CKD stage, with the highest prevalence observed among patients with stage 5 CKD (47.2%, p<0.001). Anti-HCV positivity was also significantly associated with cardiovascular disease (p=0.049), history of blood transfusion (p<0.001), and dialysis history (p<0.001). However, no significant associations were found between anti-HCV status and age (p=0.611), diabetes mellitus (p=0.445), hypertension (p=0.105), or Hb level (p=0.163).

**Table 3:** Stratification of data related to anti-HCV (total n=317) CKD: Chronic kidney disease; HCV: Hepatitis C virus; Hb: Hemoglobin

Patient variables		Anti-HCV		Pearson chi-square value	p-value
		Negative	Positive		
Gender	Female	97 (73.5%)	35 (26.5%)	20.459	<0.001
	Male	89 (48.1%)	96 (51.9%)		
Age	<52 years	92 (60.1%)	61 (39.9%)	0.258	0.611
	≥52 years	94 (57.3%)	70 (42.7%)		
Residence	Rural	87 (65.4%)	46 (34.6%)	4.291	0.038
	Urban	99 (53.8%)	85 (46.2%)		
CKD duration	<2 years	87 (65.9%)	45 (34.1%)	4.882	0.027
	≥2 years	99 (53.5%)	86 (46.5%)		
CKD severity	Stage 3	18 (81.8%)	4 (18.2%)	15.508	<0.001
	Stage 4	38 (77.6%)	11 (22.4%)		
	Stage 5	130 (52.8%)	116 (47.2%)		
Diabetes	Present	97 (56.7%)	74 (43.3%)	0.582	0.445
	Absent	89 (61.0%)	57 (39.0%)		
Hypertension	Present	154 (56.8%)	117 (43.2%)	2.632	0.105
	Absent	32 (69.6%)	14 (30.4%)		
Cardiovascular disease	Present	35 (48.6%)	37 (51.4%)	3.891	0.049
	Absent	151 (61.6%)	94 (38.4%)		
Blood transfusion history	Absent	125 (70.6%)	52 (29.4%)	23.589	<0.001
	Present	61 (43.6%)	79 (56.4%)		
Dialysis history	Absent	84 (81.6%)	19 (18.4%)	32.936	<0.001
	Present	102 (47.7%)	112 (52.3%)		
Hb	≤8 g/dl	36 (51.4%)	34 (48.6%)	1.946	0.163
	>8 g/dl	150 (60.7%)	97 (39.3%)		

Binary logistic regression analysis of anti-HCV status demonstrated that male gender (OR=0.483, 95% CI: 0.277-0.839; p=0.010), urban residence (OR=0.520, 95% CI: 0.305-0.887; p=0.016), positive history of blood transfusion (OR=0.348, 95% CI: 0.183-0.660; p=0.001), and presence of dialysis history (OR=0.211, 95% CI: 0.091-0.488; p<0.001) remained significantly associated with anti-HCV positivity after adjusting for potential confounders (Table 4). However, age (p=0.281), CKD duration (p=0.462), CKD severity (p=0.382), diabetes (p=0.839), hypertension (p=0.465), cardiovascular disease (p=0.460), and Hb level (p=0.958) were not independently associated with anti-HCV status.

**Table 4:** Binary logistic regression analysis of anti-HCV status Wald: Wald chi-square statistic; df: Degrees of freedom; CKD: Chronic kidney disease; HCV: Hepatitis C virus; Hb: Hemoglobin

Patient variables	Beta coefficient	Standard error	Wald	df	p-value	OR	95% CI for OR	
							Lower	Upper
Gender	-0.729	0.283	6.653	1	0.010	0.483	0.277	0.839
Age	-0.007	0.006	1.160	1	0.281	0.993	0.981	1.005
Residence	-0.653	0.272	5.769	1	0.016	0.520	0.305	0.887
CKD duration	0.228	0.310	0.541	1	0.462	1.256	0.684	2.308
CKD severity	-0.300	0.343	0.764	1	0.382	0.741	0.378	1.452
Diabetes	0.061	0.301	0.042	1	0.839	1.063	0.589	1.919
Hypertension	0.301	0.412	0.534	1	0.465	1.351	0.603	3.027
Cardiovascular disease	-0.265	0.359	0.545	1	0.460	0.767	0.379	1.551
Blood transfusion history	-1.056	0.327	10.459	1	0.001	0.348	0.183	0.660
Dialysis history	-1.555	0.427	13.250	1	<0.001	0.211	0.091	0.488
Hb	-0.018	0.333	0.003	1	0.958	0.983	0.512	1.886

## Discussion

The present retrospective study evaluated the frequencies of HBV and HCV infections among CKD patients in Lahore, Pakistan, and demonstrates a relatively low prevalence of HBsAg positivity (4.1%) but a markedly high prevalence of anti-HCV positivity (41.3%), indicating that HCV remains a major infectious burden among CKD patients in Pakistan. The observed prevalence of HBsAg (4.1%) is comparable to previous studies conducted among South Asian and Pakistani populations, where HBV prevalence has generally ranged between 3% and 14% [4,11,13,14]. This relatively low prevalence may reflect the impact of HBV vaccination programs, routine screening of blood products, and implementation of infection-control practices in dialysis units. In contrast, the prevalence of HCV infection in the present study (41.3%) was substantially higher than that reported in many developed countries, although it remains consistent with several regional studies from South Asia and Pakistan, where HCV prevalence among CKD and dialysis patients ranges from approximately 27% to 71% [14-17]. The high burden of HCV likely reflects repeated healthcare exposure, inadequate infection-control practices in some dialysis centers, frequent vascular access procedures, and repeated blood transfusions.

In our study, female patients demonstrated a significantly higher prevalence of HBsAg positivity than males. However, only 13 patients were HBsAg positive in our study, limiting the precision of subgroup comparisons. Furthermore, HBsAg positivity was observed exclusively among diabetic patients, resulting in a statistically significant association. Diabetes is a well-established cause of CKD and is associated with impaired immune responses, which may increase susceptibility to chronic viral infections [18,19]. Nevertheless, because of the small number of HBV-positive patients in our study, this finding should be interpreted cautiously and validated in larger studies. In contrast, HCV infection showed significant associations with several clinical and demographic characteristics, including male gender, urban residence, longer duration of CKD, advanced CKD stage, cardiovascular disease, previous blood transfusions, and dialysis history on chi-square analysis. In binary logistic regression analysis, male gender, urban residence, positive history of blood transfusion, and dialysis history persisted as independent predictors of anti-HCV positivity. These findings support existing evidence that prolonged healthcare exposure substantially increases the risk of HCV acquisition among CKD patients [4,11,16]. Patients with advanced CKD often require repeated hospitalization, vascular interventions, dialysis sessions, and blood transfusions, all of which increase opportunities for viral transmission.

The strongest associations of HCV were observed for dialysis history and blood transfusion in our study, with more than half the patients with previous dialysis or blood transfusions being anti-HCV positive. Although blood screening has improved considerably, transfusion-related transmission remains a concern in resource-limited settings such as Pakistan [9,20]. Similarly, HCV transmission within dialysis units has been attributed to lapses in standard infection-control practices, inadequate environmental disinfection, sharing of equipment, and insufficient adherence to universal precautions [21,22]. No significant association was found between HCV infection and age, diabetes, hypertension, or Hb level in our study. These findings suggest that healthcare-related exposure rather than traditional CKD risk factors may be the dominant determinant of HCV infection in this patient population.

The present study has several strengths, including inclusion of patients across different CKD stages and evaluation of multiple clinical variables associated with viral hepatitis. However, several limitations should be acknowledged. The retrospective, single-center design limits the generalizability of our findings to the broader CKD population. Anti-HCV positivity was based on serological testing without confirmation by HCV RNA PCR, making it impossible to distinguish active infection from previous exposure. Similarly, HBV DNA PCR testing was not available. The study also lacked information regarding duration and frequency of dialysis, vaccination status, previous antiviral treatment, and other potential risk factors. Additionally, the specific manufacturer and generation of the ELISA assays used for HBsAg and anti-HCV testing were not consistently documented in the retrospective laboratory records, precluding assessment of potential differences in assay performance. Furthermore, because of the observational design, causal relationships cannot be established.

Overall, the findings highlight the substantial burden of HCV among CKD patients in Pakistan and reinforce the importance of routine hepatitis screening, strict adherence to infection-prevention measures in dialysis units, safe blood transfusion practices, and timely antiviral treatment. Strengthening these preventive strategies may substantially reduce viral transmission and improve long-term outcomes among patients with CKD.

## Conclusions

Chronic hepatitis C infection was highly prevalent in CKD patients, whereas hepatitis B infection remained comparatively uncommon. Anti-HCV positivity was significantly associated with male gender, urban residence, longer CKD duration, advanced CKD stage, cardiovascular disease, previous blood transfusion, and dialysis history. A positive HBsAg result showed significant associations with female gender and diabetes. These findings emphasize the need for routine screening of HBV and HCV among CKD patients, particularly those undergoing dialysis or receiving blood transfusions. These findings underscore the importance of implementing comprehensive preventive and clinical strategies, including rigorous infection-control practices, safe blood transfusion protocols, expanded HBV immunization coverage, and early access to antiviral therapy, to reduce the impact of viral hepatitis among patients with CKD. Further multicenter prospective studies incorporating molecular testing are recommended to better define the epidemiology and determinants of hepatitis virus infections among CKD patients in Pakistan.
